# With whom to dine? Ravens' responses to food-associated calls depend on individual characteristics of the caller

**DOI:** 10.1016/j.anbehav.2014.10.015

**Published:** 2015-01

**Authors:** Georgine Szipl, Markus Boeckle, Claudia A.F. Wascher, Michela Spreafico, Thomas Bugnyar

**Affiliations:** a Department of Cognitive Biology, University of Vienna, Vienna, Austria; b Konrad Lorenz Forschungsstelle, Core Facility, University of Vienna, Gruenau im Almtal, Austria; c Department for Psychotherapy and Biopsychosocial Health, Danube University Krems, Krems, Austria; d Departamento de Ciencias Agro-Forestales, University of Valladolid, Valladolid, Spain

**Keywords:** common raven, *Corvus corax*, food-associated call, playback, recruitment

## Abstract

Upon discovering food, common ravens, *Corvus corax*, produce far-reaching ‘haa’ calls or yells, which are individually distinct and signal food availability to conspecifics. Here, we investigated whether ravens respond differently to ‘haa’ calls of known and unknown individuals. In a paired playback design, we tested responses to ‘haa’ call sequences in a group containing individually marked free-ranging ravens. We simultaneously played call sequences of a male and a female raven in two different locations and varied familiarity (known or unknown to the local group). Ravens responded strongest to dyads containing familiar females, performing more scan flights above and by perching in trees near the respective speaker. Acoustic analysis of the calls used as stimuli showed no sex-, age- or familiarity-specific acoustic cues, but highly significant classification results at the individual level. Taken together, our findings indicate that ravens respond to individual characteristics in ‘haa’ calls, and choose whom to approach for feeding, i.e. join social allies and avoid dominant conspecifics. This is the first study to investigate responses to ‘haa’ calls under natural conditions in a wild population containing individually marked ravens.

## Highlights

• Wild ravens were tested for their ability to discriminate food-associated calls.
• Calls of familiar and unfamiliar males and females were used.
• Ravens selectively approached speakers playing calls of familiar females.
• Ravens considered relative rank by preferentially approaching lower-ranking callers.

Vocalizations produced during foraging can serve various functions, for example contact calls to maintain cohesion within groups (Mahurin & Freeberg, 2009; Oda, 1996), alarm calls given by sentinels during group foraging to warn co-feeding conspecifics about danger (Manser, 1999; McGowan & Woolfenden, 1989; Wright, Berg, De Kort, Khazin, & Maklakov, 2001), and appeasement calls uttered during agonistic interactions over food to appease aggressors (Heinrich, Marzluff, & Marzluff, 1993). But acoustic signals may also be directly associated with food, indicating its location and quality (Bugnyar, Kijne, & Kotrschal, 2001; Dittus, 1984; Evans & Evans, 1999; Gros-Louis, 2006) or individual food preference (Clay & Zuberbühler, 2009; Elgar, 1986; Elowson, Tannenbaum, & Snowdon, 1991; Slocombe & Zuberbühler, 2006). Irrespective of their primary function, these different call types possibly provide receivers with cues about food availability, and attract them to feeding sites. Calls directly associated with external stimuli, such as food or predators, are termed ‘functionally referential signals’ because animals hearing these signals can respond to the referred stimulus even without seeing the actual stimulus that elicited the signal (Evans, 1997; Evans & Evans, 2007; Macedonia & Evans, 1993; Marler, Evans, & Hauser, 1992).

Recognition at the individual or class level is favoured by selection whenever it is beneficial for the signaller to be detected, and for the receiver to discriminate appropriately (Johnstone, 1997; Steiger & Müller, 2008; Tibbetts & Dale, 2007). As the benefits of signallers are not necessarily in accordance with the benefits of receivers, and, furthermore, may vary with the context, it is essential to take both context and party perspective into account when studying recognition (Tibbetts & Dale, 2007). Being individually distinct when signalling food may benefit the sender because specific individuals such as social allies may be attracted (Caine, Addington, & Windfelder, 1995). Signallers can thereby manipulate group size and composition, which can result in decreased feeding competition (Chapman & Lefebvre, 1990). Receivers could also benefit from recognizing calling individuals by assessing who is already present at the feeding site, and thus possibly predict the likelihood of competition occurring as well as receiving social support (Sharpe, Hill, & Cherry, 2013).

In ravens, three types of food-associated calls have been reported: the long ‘chii’ call uttered by juvenile ravens (Heinrich & Marzluff, 1991), the short ‘who’ call that dominant ravens utter when landing at feeding sites and the long ‘haa’ call or yell (Bugnyar et al., 2001; Heinrich & Marzluff, 1991). Most of the literature on food calls in ravens refers to the latter, the ‘haa’ call. This call type is uttered by ravens when they see food they cannot access because it is monopolized by dominant conspecifics or predators (Heinrich, 1988). Ravens are highly attracted by ‘haa’ calls of others, as previously shown in a playback study that suggests these calls may function as assembly or recruitment signals (Heinrich, 1988). Moreover, owing to their distinct morphology and context specificity, ‘haa’ calls have been hypothesized to be functionally referential (Bugnyar et al., 2001), that is, ravens hearing these calls may associate feeding opportunities with them.

A recent study revealed that ‘haa’ calls contain individually distinct features and that captive ravens were capable of discriminating between calls of two unknown individuals on the basis of these features (Boeckle, Szipl, & Bugnyar, 2012). Whether wild ravens use individual information in ‘haa’ calls in their daily lives remains untested. On the one hand, calling for an assembly at food sources could be beneficial for the sender because greater numbers of ravens might be needed to overcome monopolization by dominants (Marzluff & Heinrich, 1991), or because kin or affiliates could be among the attracted conspecifics (Braun & Bugnyar, 2012). On the other hand, differentiating between callers would allow receivers to decide whether or not to join a foraging group. These potential benefits have so far been ignored in ravens, probably because of their social organization: adult raven pairs that manage to establish a territory become breeders and defend their territories year-round (Heinrich, 1989), whereas nonbreeding birds are vagrant and tend to form relatively open groups that change in size and composition depending on the foraging situation (Heinrich, 1988). Owing to these high levels of fission–fusion dynamics in nonbreeder groups and the ephemerality of food sources in the wild, reciprocity and kin selection have been considered less important when explaining recruitment to feeding sites via ‘haa’ calls (Heinrich & Marzluff, 1991). However, long-term studies on a population of individually marked ravens in the Austrian Alps revealed that nonbreeder groups are structured by different types of social relationships, challenging the assumption of raven flocks being anonymous aggregations (Braun, Walsdorff, Fraser, & Bugnyar, 2012). Moreover, huge individual differences in vagrancy were found, with some nonbreeders being identified as local or resident to this particular valley, and others showing gradual degrees of vagrancy, visiting the valley regularly or only infrequently (Braun & Bugnyar, 2012).

Here, we tested for the recruiting function of ‘haa’ calls in the wild. Given the differences in group composition and vagrancy found in our study population, we focused on the ravens' ability to respond to food-associated calls of specific individuals and/or a particular class of individuals, respectively, by conducting simultaneous two-choice playback experiments. In each playback session, sequences of ‘haa’ calls of a male and female raven were presented simultaneously from two different locations, whereby the played-back individuals varied in the degree of familiarity to the local ravens. Different sex combinations were chosen because observations of individually marked ravens showed that females tend to call more often than males (Szipl & Bugnyar, 2014). Likewise, testing for familiarity was inspired by the observation that local nonbreeders tend to call more often than vagrant birds that only infrequently visit the study site (Szipl & Bugnyar, 2014). The playback should thus simulate a possible scenario in the birds' daily lives, that is, when they hear food-associated calls of individuals they may have repeatedly met before or of strangers that are new to this area. If receivers are able to discriminate between familiar and unfamiliar individuals on the basis of their ‘haa’ calls, they should respond to the played-back stimuli selectively, that is, approach the speaker playing back calls of familiar birds; alternatively, they could prefer to approach the speaker playing back unfamiliar birds. If ravens can discriminate familiar individuals on the basis of their regularly occurring calling activity, they should primarily approach the loudspeakers playing back ‘haa’ calls of familiar females, as females tend to produce most of the ‘haa’ calling before daily morning feedings. If they generally respond to sex, however, they should show a preference for the loudspeaker playing back ‘haa’ calls of females, irrespective of their familiarity status. As many of the birds in the study area are individually marked and subject to long-term observations, we were able to study possible effects of social knowledge (gained through repeated agonistic and affiliative interactions) on the birds' response to the playbacks. Specifically, we expected that receivers should respond to the individual stimuli selectively, that is, approach played-back calls of kin and affiliates, and avoid the speaker playing calls of opponents and birds of higher rank, respectively.

## Methods

### Study Site and Subjects

The study was conducted from February to October 2012 in the Cumberland Wildpark, a local zoo in the Northern Austrian Alps close to the village of Gruenau im Almtal (47°48′N, 13°57′E). The park attracts free-ranging ravens that forage and scrounge food from zoo animals year-round. Ravens at this site have been captured and marked in the course of long-term studies (Braun & Bugnyar, 2012; Braun et al., 2012). For this, ravens were caught in drop-in traps (Engel & Young, 1989). Traps were equipped with perches and ad libitum food and water and were checked hourly. Trapped ravens were weighed, measured (e.g. length of tarsus and beak) and ringed with an individual combination of colour rings and a metal ring containing a unique code from the German bird ringing station (Vogelwarte Radolfzell). During this standardized marking procedure, which was performed in less than 30 min by trained personnel, 50–200 μl of blood was taken from the alar vein for sexing and analysis of relatedness (for further details see Braun & Bugnyar, 2012). Age class (juvenile, subadult and adult) was estimated by the colour of the inner beak, as this changes from pink (juvenile) to black (adult) with increasing age (Heinrich & Marzluff, 1992). Frequent resightings and behavioural observations of marked birds suggest that handling and marking had no negative effects and did not elicit suspicious behaviour. Retrapping of approximately 50% of the marked ravens enabled check-ups and showed no indications of injuries (see also Boeckle et al., 2012; Braun & Bugnyar, 2012).

At the time of the study, about 200 ravens had been marked individually. Owing to the high fission–fusion dynamics that characterize ravens' social organization, the size and the composition of the population present in the valley vary over time (Braun & Bugnyar, 2012; Braun et al., 2012). The presence of marked birds was monitored during daily morning feedings (0700–0900 hours) at the enclosures of bears, *Ursus arctos*, wolves, *Canis lupus*, and wild boars, *Sus scrofa*. These enclosures were selected because ravens constantly used them and because they featured relatively open areas with a limited number of trees, allowing a good overview for human observers. The ravens were well habituated to human observers and experimental equipment (e.g. cameras, microphones) at these locations while they scrounged food from the zoo animals. Based on the amount of time spent in the valley, individual ravens were categorized according to their degree of fission–fusion dynamics and vagrancy status, respectively (Braun & Bugnyar, 2012). Those ravens that had been present on more than two-thirds of the observation days in the period before the onset of this study were labelled ‘local birds’ and considered ‘familiar’ to the ravens foraging at the enclosures. The vagrancy status of the birds whose calls were used as stimuli did not change in the course of the study. Analysis on relatedness is still ongoing, but preliminary results suggest that relatedness within this population is low, which is in line with another studied raven population (Parker, Waite, Heinrich, & Marzluff, 1994). Aside from nonbreeders, several territorial breeding pairs were known to have established territories outside the area of the park where the experiments were conducted. The study area was not within any territory boundaries, but territorial ravens frequently joined nonbreeders in the park for foraging (Drack & Kotrschal, 1995).

### Stimuli/Sound Recording and Processing

‘Haa’ calls used for the playbacks were recorded in spring 2012 in two rural areas in Austria: familiar stimuli were recorded during morning feedings at the wild boar enclosure of the Cumberland Wildpark Gruenau; unfamiliar stimuli were recorded in a captive colony of ravens located at the Haidlhof Research Station, which is about 300 km east of Gruenau. For all recordings we used a Marantz recording device (Marantz PMD-670) and a directional microphone (Sennheiser ME67/K6). Audio files (wav) had a sampling rate of 48 kHz and 16 bits amplitude resolution and were recorded at distances of 3–10 m. In a previous study, Boeckle et al. (2012) showed that ‘haa’ calls could be discriminated individually based on acoustic variables such as mean fundamental frequency, number of inflections/s (calculated as the total number of inflexions divided by call duration), amplitude modulation and harmonicity. We measured these acoustic variables in the ‘haa’ calls used as stimuli, and additionally call duration, with an automated script in Praat (www.praat.org). Only calls with a high signal-to-noise ratio and little background noise were used. For playbacks, sounds were filtered with a Hann-stop band filter between 0 and 300 Hz, and processed in Soundbooth CS4 to adjust sound pressure levels. Using the processed wav files, sequences of randomly selected ‘haa’ calls (10 calls in 2 min) of 10 subadult and adult ravens were created. This call rate resembles naturally occurring call rates of nonbreeders in the wild (Szipl & Bugnyar, 2014). In total, ‘haa’ call sequences of five males and five females were created. Of these, six sequences were of ravens familiar to the local group in Gruenau (one subadult and two adult males and one subadult and two adult females) and four were of unfamiliar ravens (one subadult and one adult male, one subadult and one adult female; Table 1).

### Playback Set-up

Paired playback experiments were conducted at the wild boar enclosure, which had a size of 5000 m^2^ and contained eight wild boars. For a schematic overview of the study area see Braun et al. (2012) and Bugnyar and Kotrschal (2001). Experiments were conducted 2 h after the morning feedings, when all the food provided to the zoo animals had been consumed and the ravens had left the feeding sites. Two speakers (Ion Block Rocker, Ion Audio, LLC. US, www.ionaudio.com; 70 Hz–50 kHz ± 3 dB) were placed on each side of the enclosure approximately 250 m apart. The experiment consisted of 10 playback sessions, with a minimum interval of 1 week between two sessions to avoid possible habituation to the experimental design. In each session, ‘haa’ call sequences of one male and one female raven were presented simultaneously and counterbalanced between speakers. After a 10 min period (termed ‘baseline phase’) without disturbances (e.g. visitors passing by, agonistic interactions e.g. chase flights of ravens, ‘haa’ calling of any raven in audible distance), the playback sequences were presented for 2 min, followed by a 10 min period after the treatment (together termed ‘treatment phase’). The 10 min period after the treatment was chosen because a previous study showed that the number of ravens arriving at a feeding site reached its peak 10 min after ‘haa’ calling had started (Bugnyar et al., 2001). Playbacks varied with respect to the familiarity of the stimuli to the local group, using the following dyads: (1) familiar male 1 versus familiar female 1; (2) familiar male 2 versus familiar female 2; (3) unfamiliar male 1 versus familiar female 3; (4) familiar male 3 versus unfamiliar female 1; and (5) unfamiliar male 2 versus unfamiliar female 2 (Table 1). Each dyad was presented twice, counterbalanced between locations of the speaker. Stimuli within a dyad were matched in call duration. Dyads were semirandomized, not allowing the same dyad to be conducted in consecutive sessions. Monitoring of the presence of marked ravens during the morning feedings as well as during the 10 min baseline phase ensured that the birds whose calls were played back were not present at the experimental site.

### Data Recording

Ravens' behaviour was observed and videotaped during the baseline and treatment phases in the periphery of both speakers and from a more distant spot overlooking the entire area. Additionally, observers in each spot ensured the identification of marked birds. ‘Haa’ calling occurs when ravens see food they cannot access (Heinrich, 1988), and ravens stay perched in trees and rarely descend on the ground before the food becomes available (Bugnyar & Kotrschal, 2001). For this reason, one of the responses measured was the number of birds that perched in the trees near the speakers (within a radius of 10 m). Ravens that hear ‘haa’ calls are attracted to the source, and have been reported to perform slow gliding or soaring flights with few or no wing beats and regular turns of the head as if observing or scanning the ground (Heinrich, 1988). Thus, another response that was measured was the frequency of scan flights directly above the speakers. Additionally, we recorded vocal responses that occurred in the vicinity of the loudspeakers. ‘Haa’ calling was scored to investigate whether ravens hearing food-associated calls would start calling as well. As Heinrich et al. (1993) showed, defensive calls uttered during aggressive interactions over food may indicate the availability of food and thereby attract ravens. Therefore, defensive calls were scored in the vicinity of the loudspeakers. Territory calls, given by territorial breeders, are long-distance calls, and thus were not included as vocal responses in the analysis as they may have been uttered further away and not as a response to the experiment. Hence, we only considered the appearance but not vocal displays of territorial breeders at the study site. The number of birds perched in the trees near the loudspeakers was recorded via scan sampling; scan flight frequency and vocal responses were measured ad libitum (Altmann, 1974).

Both behavioural and vocal responses were collected for each minute in the baseline phase (*N* = 10 sample points) and the treatment phase (*N* = 12 sample points) in both speaker positions, leading to a total of 440 data points for all 10 playback sessions conducted. Both marked and unmarked ravens were included in the data collection of behavioural and vocal responses. The identity of the marked ravens performing scan flights and perching in trees near the loudspeakers during each session was noted and analysed separately (see below).

### Behavioural Observations Outside the Experiment

In the course of this study, 35.6 ± 1 (mean ± SD) marked ravens were present at the daily morning feedings of the zoo animals. The percentage of marked birds at these feedings was approximately 60%; hence the estimated total number of ravens participating at the daily feedings was between 50 and 60 birds. Note that these birds might stay over the day or leave the park after the zoo animals were fed (Braun et al. 2012); thus, the number of birds present at the morning feeding can only be taken as a rough estimate of how many ravens could participate in our experiment.

Behavioural observations on individually marked birds were conducted during the daily morning feedings at the enclosures of bears, wolves and wild boars from January to December 2012. The behaviours observed comprised affiliative as well as agonistic interactions. Specifically, we sampled the affiliative behaviours ‘allopreening’ and ‘sitting in close contact’ using focal observations (four to seven protocols per bird, each lasting 5 min). Three of the six birds whose ‘haa’ calls were used as stimuli in the playbacks engaged in exclusive mutual affiliative behaviour with another marked individual, who was thus considered their affiliate. Additionally, behavioural observations of mild (threatening, displacement) and severe (pecking, aggressive displacement, fights) aggression were recorded ad libitum as they occur rarely and are short events. Ravens that were individually identified performing scan flights and perching in trees near the loudspeakers in the treatment phase (*N* = 10) as well as the individuals to which these marked birds responded (*N* = 3) were selected and data on initiated and received agonistic behaviours of these birds were used to calculate their ordinal dominance hierarchy (99 observations of 13 individuals). We used the modified Landau linearity index as it accounts for unknown relationships between individuals (de Vries, 1995). Ravens did not show a linear dominance hierarchy (*h* = 0.245, *P* = 0.415). Individual rank was extracted and the relative rank between birds whose calls were used as stimuli and marked responding birds was determined (‘higher’ or ‘lower’; see Table 1).

### Statistics

In a first step, we investigated whether the responses shown towards the playbacks (number of scan flights, number of birds perched in trees near the loudspeakers, number of ‘haa’ calls, and number of defensive calls) differed between the two speaker locations. Nonparametric Mann–Whitney *U* tests revealed no significant variations between speaker locations in the baseline phase (scan flights: *U* = 32.0, *N*_1_ = *N*_2_ = 10, *P* = 0.190; number of birds in trees near the loudspeakers: *U* = 25.0, *N*_1_ = *N*_2_ = 10, *P* = 0.063; number of ‘haa’ calls: *U* = 40.5, *N*_1_ = *N*_2_ = 10, *P* = 0.481; number of defensive calls: *U* = 42.5, *N*_1_ = *N*_2_ = 10, *P* = 0.579), or in the treatment phase (scan flights: *U* = 43.0, *N*_1_ = *N*_2_ = 10, *P* = 0.631; number of birds in trees near the loudspeakers: *U* = 37.0, *N*_1_ = *N*_2_ = 10, *P* = 0.353; number of ‘haa’ calls: *U* = 40.5, *N*_1_ = *N*_2_ = 10, *P* = 0.481; number of defensive calls: *U* = 42.0, *N*_1_ = *N*_2_ = 10, *P* = 0.579).

As ‘haa’ calls are uttered throughout the year, seasonal influences on ravens' responses were not expected to be strong. To investigate seasonal variation in ravens' responses, Wilcoxon signed-ranks tests were conducted and revealed no seasonal differences in the baseline phases (scan flights: *T* = −1.474, *N* = 10, *P* = 0.141; number of birds in trees near the loudspeakers: *T* = −1.583, *N* = 10, *P* = 0.113; number of ‘haa’ calls: *T* = −0.534, *N* = 10, *P* = 0.593; number of defensive calls: *T* = −0.841, *N* = 10, *P* = 0.400) and the treatment phases (scan flights: *T* = −0.867, *N* = 10, *P* = 0.386; number of birds in trees near the loudspeakers: *T* = −0.918, *N* = 10, *P* = 0.359; number of ‘haa’ calls: *T* = −0.491, *N* = 10, *P* = 0.624; number of defensive calls: *T* = −0.356, *N* = 10, *P* = 0.722).

In a second step, we calculated the difference in responses/min for each response variable between the two speaker locations for each playback session and each response variable. As speaker locations that had played female stimuli had higher mean responses than the speaker positions that had played male stimuli (see Table 1), we subtracted the responses in the speaker locations that had played male stimuli from those that had played female stimuli. These delta values (*N* = 220) of each response variable were used to calculate generalized linear mixed models (GLMMs) using the glmmADMB package version 0.7.2.12 (Fournier et al., 2012; Skaug, Fournier, Nielsen, Magnusson, & Bolker, 2012) in R version 3.0.1 (R Core Team, 2013), which allows for multiple nested random effects. For each response variable a separate GLMM was calculated with a negative binomial distribution and a log link function to account for zero-inflated count data (Zuur, Ieno, Walker, Saveliev, & Smith, 2009). To account for repeated sessions and minutes throughout the experimental phases of each dyad, a nested term was used as a random factor (1|dyad(session(experimental phase(minute)))). Familiarity pairings of the dyads (coded as F♂F♀: familiar male versus familiar female; F♂U♀: familiar male versus unfamiliar female; U♂F♀: unfamiliar male versus familiar female; U♂U♀: unfamiliar male versus unfamiliar female) and experimental phase were used as fixed factors. To rank the models, AICc values were computed, and from these the difference in AICc (ΔAICc) was calculated by subtracting the lowest AICc from all others. From this, as measures of strength of evidence for each model, the relative likelihood (exp (−0.5/ΔAICc)) and the probability or Akaike weight (relative likelihood/sum of all relative likelihoods) were computed (Burnham, Anderson, & Huyvaert, 2011). The models for all response variables are shown in Table 2. All variables and interactions that remained in the final models are presented in Table 3. Additionally, the data set was split into four subsets within the familiarity pairings, and separate models were calculated within these subsets to investigate differences between the experimental phases. As a random factor, a nested term including the sessions and minutes throughout the experimental phases (dyads F♂U♀, U♂F♀ and U♂U♀), or the sessions and minutes throughout the experimental phases within the dyads (F♂F♀) was used. Experimental phase was used as a fixed factor (Table 2).

Behavioural responses of marked individuals with respect to the relative rank of the birds whose calls were used as familiar stimuli were tested using a chi-square test.

To investigate differences in the acoustic variables of the ‘haa’ calls used as stimuli, permutated discriminant function analyses (pDFAs) with 1000 permutations and 100 random selections were conducted in R. We calculated crossed pDFAs using individual identity (*N* = 10) as the test factor and each call per raven (*N* = 10) as the control factor. Additionally, crossed pDFAs were conducted for each male–female dyad, separately, using individual identity of the male and the female within the dyad (*N* = 2) as the test factor and each call per raven (*N* = 10) as the control factor. To test for differences in call characteristics between familiar and unfamiliar ravens, we calculated nested pDFAs with familiarity (*N* = 2) as the test factor and individual identity (*N* = 10) as the control factor. To investigate whether calls differed between age classes and sexes, nested pDFAs were calculated with sex (*N* = 2) or age class (*N* = 2) as the test factor and individual identity (*N* = 10) as the control factor. As the number of variables used in pDFA should not exceed the number of test factors (Mundry & Sommer, 2007), we entered acoustic variables separately when using familiarity, sex, age class and individuals within a dyad as the test factor, and all acoustic variables at once when testing individual identity of all birds used as stimuli (see Table 4).

### Ethical Note

Trapping, marking and handling procedures of free-ranging ravens, including blood taking, were performed under licence from the Austrian Government (BMWF-66.006/0010-11/10b/2009). As the experiments were noninvasive and based on behavioural observations they do not fall under the Austrian Animal Experiments Act (§ 2, Federal Law Gazette No. 114/2012).

## Results

When simultaneously confronted with ‘haa’ calls of two different individuals of both sexes and varying familiarity in the paired playback experiment, free-ranging ravens showed differential behavioural and vocal responses (see Table 3).

### Behavioural Responses

**Figure 1:**
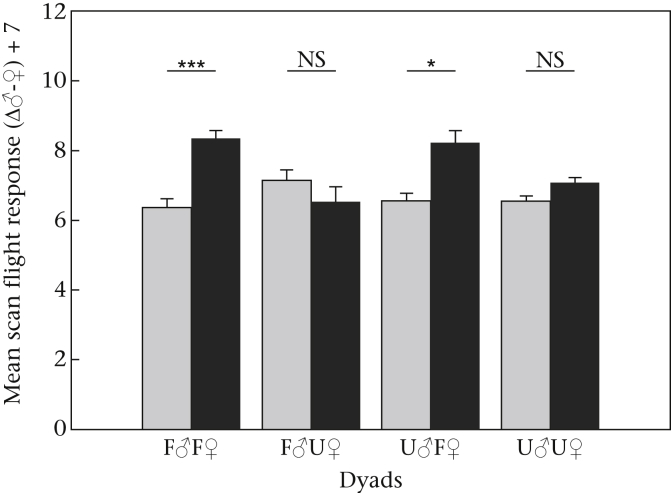
Mean number of scan flights + SE in response to ‘haa’ calls for the experimental phases ‘baseline’ (grey bars) and ‘treatment’ (black bars) for the different dyads which resemble the familiarity pairings (F = familiar to the local group; U = unfamiliar to the local group). Values represent the difference in response between the speaker that played the calls of a male and the speaker that played the calls of a female (Δ♀-♂) and we added 7 to eliminate negative values. ***P < 0.001; *P < 0.05.

The final model investigating scan flight responses included the fixed factor experimental phase, indicating the playback treatments had an effect on this response. Based on Akaike weights, the model including experimental phase, familiarity pairing and their two-way interaction explained variations in scan flight responses equally well (see Table 2), suggesting that scan flight responses were different for the different treatments applied. When split into different familiarity pairings to examine differences in the experimental phases, significantly more scan flights were performed after playing back dyads where both individuals were familiar to the local group (F♂F♀; pairwise comparison baseline versus treatment: ß = 0.268, SE = 0.081, *z* = 3.31, *P* < 0.001), and in the dyad that included an unfamiliar male and a familiar female (U♂F♀; pairwise comparison baseline versus treatment: ß = 0.231, SE = 0.114, *z* = 2.03, *P* = 0.042). No differences were found between baseline and treatment phase in the dyad where ‘haa’ calls of a familiar male and an unfamiliar female were played (F♂U♀; pairwise comparison baseline versus treatment: ß = −0.089, SE = 0.117, *z* = −0.76, *P* = 0.45), or in the dyad where both individuals were unfamiliar to the local group (U♂U♀; pairwise comparison baseline versus treatment: ß = −0.078, SE = 0.117, *z* = −0.67, *P* = 0.5; Fig. 1).

**Figure 2:**
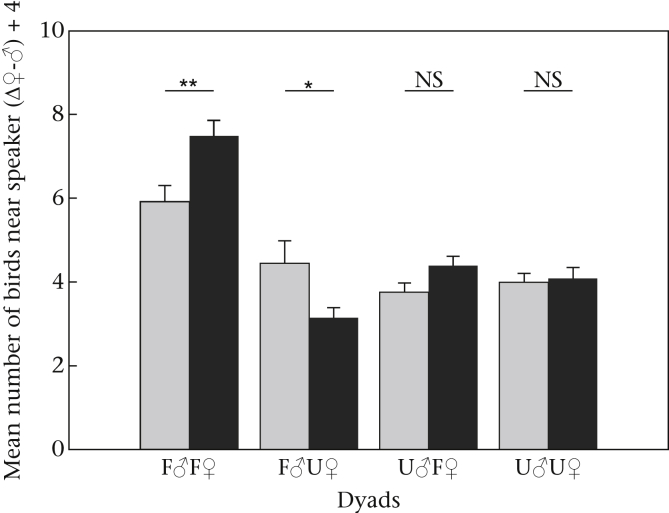
Mean number of birds perched in the trees near the loudspeakers + SE in the baseline phase (grey bars) and the treatment phase (black bars) for the dyads with varying familiarity pairings (F = familiar to the local group; U = unfamiliar to the local group). Values represent the difference in response between the speaker that played the calls of a male and the speaker that played the calls of a female (Δ♀-♂) and we added 4 to eliminate negative values. **P < 0.01; *P < 0.05.

Furthermore, the models with the highest power to explain the variation in the number of birds perched near the loudspeakers were those including the fixed factor familiarity pairing and both experimental phase and familiarity pairing (Table 2), indicating that the number of birds perched in trees differed within the experimental phases and for the different treatments applied. When the data were split into the four familiarity pairings, significantly more birds perched in trees near the speakers when both individuals in the dyad were familiar to the local group (F♂F♀; pairwise comparison baseline versus treatment: ß = 0.233, SE = 0.084, *z* = 2.76, *P* = 0.006). Significantly fewer birds perched in trees near the speaker when the playback dyad was composed of a familiar male individual and an unfamiliar female individual (F♂U♀; pairwise comparison baseline versus treatment: ß = −0.353, SE = 0.157, *z* = −2.24, *P* = 0.025). Neither the ‘haa’ call sequences of the dyad that included an unfamiliar male individual and a familiar female individual (U♂F♀; pairwise comparison baseline versus treatment: ß = 0.340, SE = 0.214, *z* = 1.59, *P* = 0.11) nor that including the two unfamiliar individuals (U♂U♀; pairwise comparison baseline versus treatment: ß = 0.041, SE = 0.213, *z* = 0.19, *P* = 0.848) had an influence on the number of birds near the speakers after the treatments (Fig. 2).

### Vocal Responses

With regard to vocal responses, no differences were found for ‘haa’ call and defensive call rates with respect to familiarity pairing and experimental phase. The models with the highest explanatory power only included the random factor, indicating that the different treatments had no effect on the frequency of ‘haa’ calls (intercept only: ß = 1.452, SE = 0.038, *z* = 38.0, *P* < 0.001) and defensive calls (intercept only: ß = 2.597, SE = 0.024, *z* = 108.0, *P* < 0.001).

### Identity of Responding Birds

When both stimuli in a playback session were of individuals that were familiar to the local group, marked birds tended to approach the speaker that was playing calls of ravens lower in rank than themselves (chi-square: χ^2^_1_ = 3.6, *N* = 10, expected probability = 0.5, *P* = 0.058; Table 1). With regard to relatedness and affiliative behaviours, three birds whose ‘haa’ calls were used as familiar stimuli were known to engage in reciprocal affiliations with marked ravens. Anecdotally, two marked males approached the speakers that played ‘haa’ call sequences of their female affiliates. This was not the case for the speakers playing stimuli of the marked familiar male (*N* = 1).

### Acoustic Analysis

The results of the pDFA showed that ‘haa’ calls differed significantly between individuals (*P* = 0.001) when using the acoustic variables call duration, mean fundamental frequency (mean F0), number of inflections/s, amplitude modulation and harmonicity (compare also Boeckle et al., 2012). None of these acoustic parameters, however, were sufficient to classify calls of individuals based on familiarity, sex or age class (see Table 4). This indicates that ravens' responses were unlikely to be based on acoustic differences on a class level (familiarity, sex or age class), but were based on individual vocal characteristics. Classification of individuals within each dyad was achieved using mean fundamental frequency (all dyads), amplitude modulation (dyad 2 and dyad 3) and harmonicity (dyad 2, dyad 3 and dyad 4; see Table 4).

## Discussion

This study provides the first evidence for differential responses to food-associated calls of particular individuals in a group of free-ranging common ravens. Ravens responded strongest to playbacks of dyads with individuals that were familiar to the local group and, within these, tended to prefer stimuli of female individuals over stimuli of males.

### Behavioural Responses

The most prominent behavioural responses to the playbacks were scan flights over the area around the speaker, which significantly increased after the playback of ‘haa’ call sequences in general. This finding confirms the recruitment function of ‘haa’ calls (Heinrich, 1988) and indicates a biologically meaningful response to the playback, as ravens typically perform scan flights when searching for food. Furthermore, it supports the idea of the referential function of ‘haa’ calls (Bugnyar et al., 2001): hearing the call sequences in our experiment elicited not only the approach of receivers (recruitment), but also the behaviours indicative of an expectation of available food or the presence of a calling individual that had sighted food.

Most scan flights were performed after we played ‘haa’ calls of two familiar individuals and of an unfamiliar male and a familiar female (dyads F♂F♀ and U♂F♀); however, playing calls of a familiar male and an unfamiliar female did not elicit a significant response. These findings support our assumption that familiarity of the calling individual plays a role in the receivers' decision of whether or not to approach the speaker. Yet, familiarity does not explain the full picture, as a familiar male was treated differently from a familiar female but similarly to an unfamiliar female. This pattern is corroborated by our second behavioural parameter, the number of birds perched in trees near the speakers: while more birds perched in the trees after we played the calls of two familiar individuals, fewer birds did so after we played the calls of a familiar male and a unfamiliar female. Taken together, these results speak in favour of receivers responding to specific (familiar) individuals, preferably females. The reason for responding more strongly to females may be that females are lower in rank than males (Braun & Bugnyar, 2012), and produce ‘haa’ calls more often (Szipl & Bugnyar, 2014), probably to gain social support.

Class level recognition based on familiarity or sex may have caused these differential responses. Recognition based on familiarity is thought to be the underlying mechanism in the so called ‘dear enemy effect’ (Fisher, 1954), where individuals reduce aggression towards neighbours compared to strangers. Within corvids, Mexican jays, *Aphelocoma ultramarina*, were shown to be able to discriminate strangers from members of their stable social group via primary calls (Hopp, Jablonski, & Brown, 2001). In this respect, it is noteworthy that nonbreeder ravens do not live in stable social groups but show high levels of fission–fusion dynamics, which probably increases the cognitive load in dealing with others (Aureli et al., 2008). Furthermore, raven ‘haa’ calls are described as common and widespread discrete calls (Pfister, 1988), which suggests that ravens in our study discriminated familiar from unfamiliar individuals based on learned individual cues, not by regional or group-specific differences or dialects. Sex differences in vocalizations are usually caused by morphological differences in size, and are expressed by differences in the fundamental frequency of the calls (Fitch, 1997). A preliminary study on the acoustic structure of ‘haa’ calls (Boeckle, Szipl, & Bugnyar, n.d.) found a significant effect of age but not of sex, which might be explained by the maturation of the vocal tract and the relatively minor differences in body size between the sexes of adult birds. In our study, dyads used for playbacks were matched in age class, and ‘haa’ calls of individuals could in theory be classified by age class based on call duration (Boeckle et al., n.d.); however, this was controlled for within the dyads.

The sound analysis of the stimuli used for the playbacks in the current study strongly supports the notion of individual features derived from previous studies: whereas the pDFA failed to discriminate the ‘haa’ calls used as stimuli based on the measured acoustic variables when grouped for sex and familiarity, it was highly significant at an individual level. Individuals were correctly classified using mean fundamental frequency, amplitude modulation and harmonicity, confirming the results of Boeckle et al. (2012). The fact that ‘haa’ calls in the tested population were individually distinct, and that captive ravens were shown to discriminate between them (Boeckle et al., 2012) suggests that responding ravens in this study used individual-specific rather than unidentified class-specific cues when responding differently to familiar and unfamiliar ‘haa’ calls of male and female individuals of different age classes. In addition, within the dyad that contained stimuli of two unfamiliar individuals, the number of scan flights and birds perched in trees did not differ for the male and the female individual, which points to recognition on an individual level with learned individual-specific cues about the sender (Tibbetts & Dale, 2007). Our sample size is small, however, and, as the study was conducted under field conditions, we cannot exclude the possibility that some of the ravens were attracted by the behaviour of some other individuals (flying in one direction or perching in specific trees). Local enhancement would thus be a possible underlying mechanism that could explain some of the results.

The assumption that responding ravens may have used individual-specific acoustic cues rather than class-specific cues receives further support when we examine the identity of marked responding birds: there was a tendency for responding birds to approach the speakers that played calls of ravens lower in rank than themselves. Only two of 10 birds responded to calls of ravens higher in rank than themselves, and these two juvenile ravens were lowest in rank, hence they could not respond to calls of birds lower in rank than themselves. With respect to different sexes, nine of the 10 birds responded to females, and only one to a male individual (see Table 1). Furthermore, within the dyads that contained two familiar stimuli, we can anecdotally report two males approaching the speakers that played calls of their female affiliates. Ravens thus seemingly recognized the calls of those familiar individuals whose calls were used in the playback experiment, and associated them with past interactions when deciding whether or not to join a calling conspecific. A similar result was found in dwarf mongooses, *Helogale parvula*, which are more vigilant to calls of higher-ranking conspecifics that could steal their food (Sharpe et al., 2013). The effect of rank may also explain why stimuli of female individuals were preferred over males: as females are usually lower in rank (Braun & Bugnyar, 2012), a possible interaction of rank and sex could explain this result, but this needs to be tested with a larger sample size and preferably also in other populations.

### Vocal Responses

In terms of vocal responses, hardly any yelling was recorded, showing that ‘haa’ calls were not contagious, and did not elicit calling in the absence of food. This is in sharp contrast to many studies investigating food-associated calls in primates, where food calls usually elicit food calls as a response (Gros-Louis, 2004; Roush & Snowdon, 2000; Schel, Machanda, Townsend, Zuberbühler, & Slocombe, 2013). This finding stresses the need to investigate the diverse functions of food-associated calls in different species in more detail, as the term ‘food-associated call’ implies a common function which still needs to be tested (Clay, Smith, & Blumstein, 2012). The number of defensive calls was also low and did not differ between the baseline and the treatment phase. Defensive calls may occur when ravens fight over food, and hence could be used by ravens to detect food sources, as was shown for begging calls (Heinrich et al., 1993). This strengthens the result that attraction to the speakers was not due to any other cues given, but in consequence of the stimuli played.

Adult raven pairs that manage to establish a territory become highly dominant breeders, and both territory holders and nonbreeders gather at feeding events (Heinrich, 1988; Webb, Marzluff, & Hepinstall-Cymerman, 2012). One territorial pair was observed on one of the 10 playback sessions within the study area. This pair appeared directly after the presentation of the dyad in which both individuals were unfamiliar; however, they did not perform scan flights nor did they perch in trees near either of the speakers, and thus they were not included in the analysis. As ‘haa’ calls are mainly used by nonbreeding individuals for recruitment (Heinrich, 1988), territorial breeders might show a territorial response upon hearing ‘haa’ calls to prevent large numbers of individuals being attracted to the feeding site. Dyads including stimuli of familiar male or female individuals did not elicit the attendance of territorial birds, suggesting a ‘dear enemy’ effect (Fisher, 1954), where familiarity reduces aggression, the time and energy spent fighting and the risk of injuries. Adult ravens have low mortality rates (Holyoak, 1971). Excluding accidents and shooting, injuries caused by conspecifics are one of the main causes of death in this species (Szipl, Loretto, & Bugnyar, n.d.). For that very reason, competition and aggressive interactions over food are very costly and might have favoured the evolution of individual recognition in ravens from the receiver side. Alternatively, individual recognition could be a domain-general ability in ravens that is also used to avoid aggression and/or reduce competition over food.

We stress that our approach of studying free-ranging ravens that exploit the food of zoo animals has its constraints and we need to be careful in generalizing our findings to ravens in other ecological conditions. However, as typical scavengers, ravens are adapted to make use of resources provided by heterospecifics (Marzluff & Angell, 2005), which in a human-influenced environment includes using agricultural areas (Enggist-Dueblin & Pfister, 2002), rubbish dumps (Boarman, Patten, Camp, & Collis, 2006; Webb et al., 2012) and facilities with livestock (Wright, Stone, & Brown, 2003) and game (Ratcliffe, 1997). In this respect, our study population seems to be the norm rather than the exception. So far, all our empirical data indicate that it is representative for the alpine situation in Central Europe.

Taken together, the current study provides the first experimental test for raven social knowledge under field conditions. Contrary to the predominant assumption that foraging groups represent largely anonymous crowds (Heinrich, 1988), our findings are in line with the idea that at least the studied population represents a highly structured and individualized society within nonbreeders. Because of the social organization, characterized by dynamic group composition and size, the ability to selectively respond to food-associated ‘haa’ calls of specific individuals seems a remarkable cognitive performance. It probably requires memorization of distinct features of a large number of birds as well as their relative social status. Our findings suggest that the usage and understanding of food-associated calls are highly complex and that ravens are able to tactically use gathered information. Hence, future studies should focus in more detail on the group composition and how it relates to individual calling activity in wild ravens.

## Figures and Tables

**Table 1:** Information about playback design and individuals of which calls were used as stimuli and responding birds

Dyad	Session	Speaker	Playback stimulus				Mean number of responding ravens±SD		Individual information on marked ravens performing scan flights and perching in trees near the speakers							
			ID	Age class	Sex	Familiarity	Scan flight	Perched near speaker	Age class			Sex		Relative rank		
									Juvenile	Subadult	Adult	Male	Female	Higher	Lower	Kin/ Affiliate
(1)	1	1	Ki	Subadult	M	Familiar	2.25±2.86	1.92±0.51	1	0	0	0	1	0	1	0
(1)	1	2	La	Subadult	F	Familiar	2.33±2.46	5.83±1.03	0	0	3	1	2	3	0	0
(1)	2	1	La	Subadult	F	Familiar	1.50±1.51	3.58±0.67	0	0	3	2	1	3	0	0
(1)	2	2	Ki	Subadult	M	Familiar	1.33±2.06	0	0	0	0	0	0	0	0	0
(2)	1	1	Ti	Adult	F	Familiar	1.42±2.07	0.42±0.51	0	0	1	1	0	0	0	1
(2)	1	2	Mr	Adult	M	Familiar	0.17±0.39	0.42±0.51	0	0	0	0	0	0	0	0
(2)	2	1	Mr	Adult	M	Familiar	1.58±1.24	0.17±0.39	0	0	0	0	0	0	0	0
(2)	2	2	Ti	Adult	F	Familiar	3.08±1.78	6.58±1.93	1	0	1	1	1	1	1	0
(3)	1	1	Ma	Subadult	M	Unfamiliar	0.17±0.39	0	0	0	0	0	0	–	–	–
(3)	1	2	Bi	Subadult	F	Familiar	1.58±1.56	0.92±1.08	0	0	1	1	0	0	0	1
(3)	2	1	Bi	Subadult	F	Familiar	1.50±1.93	1.00±0.95	0	0	1	0	1	1	0	0
(3)	2	2	Ma	Subadult	M	Unfamiliar	0.58±0.9	0.83±1.03	0	0	0	0	0	–	–	–
(4)	1	1	Ca	Adult	M	Familiar	0.17±0.39	3.25±0.97	0	0	0	0	0	0	0	0
(4)	1	2	He	Adult	F	Unfamiliar	0.67±0.98	1.75±1.06	0	0	0	0	0	–	–	–
(4)	2	1	He	Adult	F	Unfamiliar	0.42±0.79	0.17±0.58	0	0	0	0	0	–	–	–
(4)	2	2	Ca	Adult	M	Familiar	1.83±1.99	0.42±0.79	0	0	0	0	0	0	0	0
(5)	1	1	Jo	Adult	F	Unfamiliar	0.92±1.44	1.58±0.51	0	0	0	0	0	–	–	–
(5)	1	2	Ja	Adult	M	Unfamiliar	0.75±0.75	2.33±0.49	0	0	0	0	0	–	–	–
(5)	2	1	Ja	Adult	M	Unfamiliar	0	0	0	0	0	0	0	–	–	–
(5)	2	2	Jo	Adult	F	Unfamiliar	0	0.92±1.0	0	0	0	0	0	–	–	–

Age class, sex (M = male, F = female), familiarity with regard to the tested local group and mean number of scan flights and birds perched in trees ± SD in the treatment phase are provided for each individual whose calls were used as stimuli. Additionally, individual information on marked birds with known identity is provided and summarized for the number of scan flights and birds perched in trees near the speakers, their age class and sex and their relative rank to the birds whose calls were used as stimuli.

**Table 2:** Model selection for response variables from generalized linear mixed models

Variable	Random factor	Model	AICc	ΔAICc	Relative likelihood	Akaike weight
Scan flights	(1|dyad/session/phase/min)	Familiarity pairing* Experimental phase (Full model)	**933.120**	**1.981**	**0.371**	**0.227**
		Familiarity pairing+Experimental phase	934.735	3.596	0.166	0.101
		Familiarity pairing	940.415	9.276	0.010	0.006
		**Experimental phase**	**931.139**	**0**	**1.000**	**0.610**
		Intercept only	935.894	4.755	0.093	0.057
Number of birds perched in trees near loudspeakers	(1|dyad/session/phase/min)	Familiarity pairing* Experimental phase (Full model)	875.822	4.466	0.107	0.052
		**Familiarity pairing+Experimental phase**	**872.553**	**1.642**	**0.440**	**0.215**
		**Familiarity pairing**	**870.807**	**0**	**1.000**	**0.488**
		Experimental phase	873.237	2.574	0.276	0.135
		Intercept only	873.584	2.962	0.227	0.111
‘Haa’ calls	(1|dyad/session/phase/min)	Familiarity pairing* Experimental phase (Full model)	838.646	11.086	0.004	0.002
		Familiarity pairing+Experimental phase	832.241	5.126	0.077	0.047
		Familiarity pairing	830.137	3.126	0.210	0.127
		Experimental phase	828.893	2.026	0.363	0.220
		**Intercept only**	**826.826**	**0**	**1.000**	**0.605**
Defensive calls	(1|dyad/session/phase/min)	Familiarity pairing* Experimental phase (Full model)	1191.620	10.674	0.005	0.004
		Familiarity pairing+Experimental phase	1187.525	6.024	0.049	0.037
		Familiarity pairing	1185.683	4.286	0.117	0.088
		Experimental phase	1184.991	3.738	0.154	0.116
		**Intercept only**	**1181.212**	**0**	**1.000**	**0.754**

Bold type indicates the best models, which were determined based on relative AICc values (ΔAICc) and computed relative likelihood and Akaike weights.

**Table 3:** Values of final models derived from GLMMs for all response variables

Variable	Subset	Final model	Coefficients	Estimate	SE	CI (2.5%)	CI (97.5%)	*z*	*P*
Scan flights		Experimental phase	Intercept	1.886	0.041	1.805	1.964	45.47	<0.001
			Experimental phase (baseline versus treatment)	0.155	0.055	0.048	0.262	2.84	<0.005
	F♂F♀		Intercept	1.852	0.066	1.722	1.982	27.92	<0.001
			Experimental phase (baseline versus treatment)	0.268	0.081	0.109	0.426	3.31	<0.001
	F♂U♀		Intercept	1.962	0.113	1.741	2.183	17.39	<0.001
			Experimental phase (baseline versus treatment)	−0.089	0.117	−0.317	0.140	−0.76	0.450
	U♂F♀		Intercept	1.880	0.088	1.707	2.052	21.34	<0.001
			Experimental phase (baseline versus treatment)	0.231	0.114	0.008	0.453	2.03	0.042
	U♂U♀		Intercept	1.880	0.008	1.707	2.052	21.34	<0.001
			Experimental phase (baseline versus treatment)	0.078	0.117	−0.151	0.308	0.67	0.500
Number of birds perched in trees near loudspeaker		Familiarity pairing	Intercept	1.853	0.115	1.627	2.079	16.07	<0.001
			F♂F♀ versus F♂U♀ (F♂F♀=0, F♂U♀=1)	−0.569	0.206	−0.972	−0.165	−2.76	0.006
			F♂F♀ versus U♂F♀ (F♂F♀=0, U♂F♀=1)	−0.469	0.204	−0.869	−0.068	−2.29	0.022
			F♂F♀ versus U♂U♀ (F♂F♀=0, U♂U♀=1)	−0.478	0.205	−0.879	−0.077	−2.34	0.019
			F♂U♀ versus U♂F♀ (F♂U♀=0, U♂F♀=1)	0.100	0.240	−0.371	0.571	0.42	0.677
			F♂U♀ versus U♂U♀ (F♂U♀=0, U♂U♀=1)	0.091	0.240	−0.380	0.562	0.38	0.706
			U♂F♀ versus U♂U♀ (U♂F♀=0, U♂U♀=1)	−0.010	0.239	−0.478	0.459	−0.04	0.968
	F♂F♀		Intercept	1.738	0.166	1.413	2.063	10.48	<0.001
			Experimental phase (baseline versus treatment)	0.233	0.084	0.067	0.398	2.76	0.006
	F♂U♀		Intercept	1.493	0.107	1.284	1.702	14.01	<0.001
			Experimental phase (baseline versus treatment)	−0.353	0.157	−0.662	−0.045	−2.24	0.025
	U♂F♀		Intercept	0.524	0.256	0.023	1.023	2.05	0.040
			Experimental phase (baseline versus treatment)	0.340	0.214	−0.079	0.760	1.59	0.110
	U♂U♀		Intercept	0.618	0.324	−0.016	1.252	1.91	0.056
			Experimental phase (baseline versus treatment)	0.041	0.213	−0.376	0.458	0.19	0.848
‘Haa’ calls		Intercept only	Intercept	1.452	0.038	1.377	1.527	38.0	<0.001
Defensive calls		Intercept only	Intercept	2.597	0.024	2.550	2.644	108.0	<0.001

CI = confidence interval, *z* = effect size. For experimental phase, baseline = 0 and treatment = 1.

**Table 4:** Results of crossed and nested pDFA for raven stimuli used in the playback experiments

Test factor	*N* test	Control factor	*N* control	Acoustic variable	*N* correct	*P*
Familiarity	2	Individual identity	10	Duration	65	0.093
Familiarity	2	Individual identity	10	Mean F0	59	0.477
Familiarity	2	Individual identity	10	Number of inflections/s	61	0.160
Familiarity	2	Individual identity	10	Amplitude modulation	60	0.987
Familiarity	2	Individual identity	10	Harmonicity	73	0.164
Sex	2	Individual identity	10	Duration	49	1.000
Sex	2	Individual identity	10	Mean F0	55	0.624
Sex	2	Individual identity	10	Number of inflections/s	53	0.441
Sex	2	Individual identity	10	Amplitude modulation	70	0.431
Sex	2	Individual identity	10	Harmonicity	59	0.697
Age class	2	Individual identity	10	Duration	78	0.042
Age class	2	Individual identity	10	Mean F0	59	0.496
Age class	2	Individual identity	10	Number of inflections/s	61	0.146
Age class	2	Individual identity	10	Amplitude modulation	58	0.445
Age class	2	Individual identity	10	Harmonicity	73	0.181
Individual identity	10	Calls	10	Duration+Mean F0+Number of inflections/s+Amplitude modulation+Harmonicity	88	0.001
Dyad 1 (La+Ki)	2	Calls	10	Duration	14	0.228
Dyad 1 (La+Ki)	2	Calls	10	Mean F0	19	0.005
Dyad 1 (La+Ki)	2	Calls	10	Number of inflections/s	12	0.495
Dyad 1 (La+Ki)	2	Calls	10	Amplitude modulation	12	0.588
Dyad 1 (La+Ki)	2	Calls	10	Harmonicity	15	0.056
Dyad 2 (Ti+Mr)	2	Calls	10	Duration	10	0.980
Dyad 2 (Ti+Mr)	2	Calls	10	Mean F0	18	0.006
Dyad 2 (Ti+Mr)	2	Calls	10	Number of inflections/s	13	0.246
Dyad 2 (Ti+Mr)	2	Calls	10	Amplitude modulation	20	0.001
Dyad 2 (Ti+Mr)	2	Calls	10	Harmonicity	18	0.009
Dyad 3 (Bi+Ma)	2	Calls	10	Duration	13	0.259
Dyad 3 (Bi+Ma)	2	Calls	10	Mean F0	20	0.002
Dyad 3 (Bi+Ma)	2	Calls	10	Number of inflections/s	11	1.000
Dyad 3 (Bi+Ma)	2	Calls	10	Amplitude modulation	20	0.002
Dyad 3 (Bi+Ma)	2	Calls	10	Harmonicity	16	0.025
Dyad 4 (He+Ca)	2	Calls	10	Duration	11	0.882
Dyad 4 (He+Ca)	2	Calls	10	Mean F0	16	0.029
Dyad 4 (He+Ca)	2	Calls	10	Number of inflections/s	10	0.926
Dyad 4 (He+Ca)	2	Calls	10	Amplitude modulation	20	0.005
Dyad 4 (He+Ca)	2	Calls	10	Harmonicity	15	0.065
Dyad 5 (Jo+Ja)	2	Calls	10	Duration	11	0.909
Dyad 5 (Jo+Ja)	2	Calls	10	Mean F0	18	0.015
Dyad 5 (Jo+Ja)	2	Calls	10	Number of inflections/s	11	0.908
Dyad 5 (Jo+Ja)	2	Calls	10	Amplitude modulation	14	0.127
Dyad 5 (Jo+Ja)	2	Calls	10	Harmonicity	19	0.007

*N* test and *N* control denote the number of test and control factors, respectively. *N* correct denotes the number of correctly classified calls.
